# Development and internal validation of a nomogram-based screening tool for concurrent autoimmune hepatitis in children with intestinal diseases: a retrospective cohort study

**DOI:** 10.3389/fmed.2026.1861363

**Published:** 2026-10-07

**Authors:** YanFang Hou

**Affiliations:** Department of Pediatric Ward One, Kaifeng Central Hospital, Kaifeng, Henan, China

**Keywords:** autoimmune hepatitis, children, intestinal disease, nomogram, risk stratification, screening model

## Abstract

**Background:**

Autoimmune hepatitis (AIH) can complicate paediatric intestinal diseases, but practical tools for early screening and risk stratification are limited.

**Methods:**

This retrospective cohort study included 346 children with intestinal diseases treated at a tertiary centre from 2015 to 2023, including 68 with concurrent AIH and 278 without AIH. Baseline clinical and laboratory variables measured at the initial gastroenterology evaluation were analysed. Candidate variables were selected using least absolute shrinkage and selection operator logistic regression and entered into multivariable logistic regression to construct a nomogram-based screening model. Model performance was assessed using receiver operating characteristic curve analysis, calibration metrics, bootstrap internal validation, and decision curve analysis.

**Results:**

Eight variables associated with concurrent AIH were included: disease duration ≥12 months, C-reactive protein ≥15 mg/L, immunoglobulin G ≥16 g/L, erythrocyte sedimentation rate ≥25 mm/h, antinuclear antibody positivity, corticosteroid use, pancolitis, and family history of autoimmune disease. The model showed an apparent area under the curve (AUC) of 0.974 and a bootstrap-corrected AUC of 0.893. Calibration was acceptable, with a slope of 0.87, intercept of −0.03, Brier score of 0.098, and a non-significant Hosmer–Lemeshow test (*p* = 0.398). Decision curve analysis indicated a greater net benefit for referral-oriented screening than single-predictor or default strategies.

**Conclusion:**

The nomogram may help screen children with intestinal diseases who warrant early hepatological evaluation for concurrent AIH. It should not be used as a standalone diagnostic instrument, and external validation is required before routine clinical use.

## Introduction

Autoimmune hepatitis (AIH) is a chronic, immune-mediated inflammatory liver disease characterised by elevated transaminases, hypergammaglobulinemia, circulating autoantibodies, and interface hepatitis on histology (1). In paediatric patients, AIH may present with a wide clinical spectrum ranging from asymptomatic liver enzyme elevation to fulminant hepatic failure (2). Although AIH can occur in isolation, accumulating evidence indicates a significant association between AIH and intestinal diseases, particularly inflammatory bowel disease (IBD) (3, 4).

The gut–liver axis represents a bidirectional communication network linking the gastrointestinal tract and the liver through the portal venous system, bile acids, and immune signalling (5). In children with intestinal diseases, chronic intestinal inflammation may increase intestinal permeability, allowing microbial antigens and pro-inflammatory mediators to translocate to the liver via portal circulation, potentially triggering hepatic autoimmunity in genetically susceptible individuals (6, 7). Epidemiological studies report that the prevalence of AIH among children with IBD ranges from 1.5 to 7.5%, substantially exceeding the prevalence in the general paediatric population (8, 9).

The early identification of children with intestinal diseases who are at risk for AIH is clinically important, as timely diagnosis and treatment can prevent progression to fibrosis, cirrhosis, and liver failure (10, 11). However, the diagnosis of AIH in the context of intestinal disease is often delayed because hepatic symptoms may be masked by predominant gastrointestinal manifestations, and mildly elevated liver enzymes may be attributed to drug hepatotoxicity or disease activity rather than autoimmune hepatitis (12). To date, no validated clinical screening or risk-stratification tool exists for systematically identifying children with intestinal diseases who warrant hepatological evaluation for concurrent AIH risk (13).

Nomograms are graphical representations of multivariable risk models that enable clinicians to calculate individualised risk estimates at the bedside (14). They have been widely applied in oncology and, increasingly, in hepatology for diagnostic and prognostic purposes (15). The purpose of this study is to develop and internally validate a nomogram-based screening and risk-stratification model for identifying children with intestinal diseases who warrant further evaluation for concurrent AIH, using routinely available clinical and laboratory parameters collected at the initial gastroenterology evaluation.

## Methods

### Study design and participants

Formal ethical review and individual informed consent were not required because this retrospective study used anonymised clinical data. The study was conducted in accordance with the Declaration of Helsinki, and all patient data were anonymised before analysis. A total of 346 children (age <18 years) diagnosed with intestinal diseases, including IBD (ulcerative colitis [UC], Crohn’s disease, and IBD-unclassified), coeliac disease, and other specified intestinal conditions, were enrolled from a single tertiary paediatric centre between January 2015 and December 2023.

Eligibility criteria included: (1) a confirmed diagnosis of intestinal disease based on established clinical, endoscopic, and histological criteria; (2) availability of baseline clinical and laboratory data sufficient for analysis at the initial gastroenterology evaluation; (3) subsequent hepatological assessment performed during follow-up. Exclusion criteria included: (1) a pre-existing diagnosis of chronic liver disease prior to the onset of intestinal disease; (2) evidence of viral hepatitis (hepatitis A, B, C, or E), Wilson disease, alpha-1 antitrypsin deficiency, or drug-induced liver injury at any time during follow-up; (3) more than 20% missing data among the core study variables.

### Predictor assessment and timing

All candidate predictor variables were measured at the time of initial gastroenterology evaluation, defined as the first outpatient or inpatient encounter at which the intestinal disease was diagnosed, and a standardised laboratory panel was obtained. This baseline assessment preceded any hepatological referral or AIH-specific workup. Specifically, demographic data (age, sex, body mass index *z*-score), disease characteristics (type and duration of intestinal disease, extent of colonic involvement, extraintestinal manifestations, family history of autoimmune disease), medication exposure (5-aminosalicylic acid, corticosteroids, thiopurines, methotrexate, biologic agents, exclusive enteral nutrition), and laboratory parameters (alanine aminotransferase [ALT], aspartate aminotransferase [AST], gamma-glutamyl transferase [GGT], alkaline phosphatase [ALP], total bilirubin, albumin, immunoglobulin G [IgG], C-reactive protein [CRP], erythrocyte sedimentation rate [ESR], faecal calprotectin, prothrombin time–international normalised ratio [PT-INR], antinuclear antibody [ANA], and smooth muscle antibody [SMA]) were extracted from the medical records corresponding to the above-noted initial visit.

### Outcome definition

The primary outcome was the presence of concurrent AIH, diagnosed according to the International Autoimmune Hepatitis Group (IAIHG) simplified criteria (16), supplemented by expert hepatologist adjudication. The diagnosis of AIH was established during subsequent hepatological assessment triggered by persistent or progressive liver enzyme abnormalities identified during routine monitoring of the intestinal disease. The temporal sequence was thus: initial gastroenterology evaluation (predictor measurement) followed by hepatological workup (outcome ascertainment). Among the 68 children with AIH, the median interval from the initial gastroenterology evaluation to AIH diagnosis was 8.2 months (interquartile range [IQR] 4.5–15.8 months).

### Statistical analysis

Continuous variables were expressed as mean ± standard deviation (SD) or median (IQR), depending on the distribution assessed by the Shapiro–Wilk test. Categorical variables were presented as frequencies and percentages. Between-group comparisons were performed using the independent-samples *t*-test or Mann–Whitney *U* test for continuous variables and the chi-squared test or Fisher’s exact test for categorical variables.

Variables with *p* < 0.10 in the univariate analysis were entered into a least absolute shrinkage and selection operator (LASSO) logistic regression for variable selection, using 10-fold cross-validation to determine the optimal regularisation parameter (*λ*) at one standard error above the minimum deviance (*λ*_1se_). This approach was chosen over forward stepwise selection because LASSO provides simultaneous variable selection and coefficient shrinkage, reducing the risk of overfitting and producing more stable variable selection in the setting of correlated predictors (17). The variables retained by LASSO with non-zero coefficients were then entered into a standard multivariable logistic regression model to obtain unpenalised odds ratios (ORs) and 95% confidence intervals (CIs) for clinical interpretability.

Certain continuous biomarkers (disease duration, CRP, IgG, and ESR) were dichotomised for the final model to enhance clinical usability. The cutoff values were determined using a combined clinical and data-driven approach. Receiver operating characteristic curve (ROC) analysis was used to identify thresholds with the highest combined sensitivity and specificity according to the Youden index, and these thresholds were then considered in relation to commonly used clinical reference values. Because data-driven thresholds may be sample-dependent, these cutoffs were used to support practical screening rather than to define diagnostic criteria. Disease duration was dichotomised at 12 months, based on the clinical rationale that sustained intestinal inflammation beyond 1 year reflects chronic immunological activation, and the corresponding ROC curve analysis confirmed this as the optimal discriminatory threshold (area under the curve [AUC] 0.623, Youden index 0.218). For CRP (cutoff 15 mg/L; AUC 0.752, Youden index 0.445), IgG (cutoff 16 g/L; AUC 0.882, Youden index 0.662), and ESR (cutoff 25 mm/h; AUC 0.734, Youden index 0.408), the selected cutoffs closely corresponded to established clinical reference thresholds and yielded the highest combined sensitivity and specificity. Sensitivity analyses using continuous forms of these variables yielded associations in the same direction and with consistent statistical significance as the dichotomised analyses.

A nomogram was constructed based on the final multivariable logistic regression model to visualise the estimated screening risk of concurrent AIH for individual patients. Discrimination was assessed by the AUC of the ROC curve. The model’s apparent AUC was calculated using the entire development cohort. Internal validation was performed using 1,000 bootstrap resamples to estimate the optimism-corrected AUC and to derive a uniform shrinkage factor. The difference between the apparent AUC and the corrected AUC represents the estimated optimism, reflecting the expected decrease in discriminative performance when the model is applied to new data.

Calibration was evaluated using multiple approaches. The Hosmer–Lemeshow goodness-of-fit test assessed overall calibration. Additionally, a calibration plot was generated comparing observed and estimated probabilities across deciles. The calibration slope and calibration intercept were estimated from a logistic recalibration model, where a slope of 1.0 and an intercept of 0.0 indicated perfect calibration. The Brier score, representing the mean squared difference between the observed outcomes and estimated probabilities, was calculated as an overall measure of risk-estimation accuracy, with lower values indicating better performance (18).

Decision curve analysis (DCA) was performed to evaluate the clinical utility of the nomogram by plotting the net benefit against threshold probabilities, comparing the nomogram with single-predictor strategies and default strategies of treating all or treating none (19).

Logistic regression rather than a time-to-event model was selected as the analytical framework because the primary clinical objective is to estimate the probability of concurrent (prevalent) AIH at the time of intestinal disease evaluation, rather than to predict the time to future AIH onset. In this cohort, AIH was identified through standardised hepatological assessment triggered by laboratory abnormalities during follow-up, and the exact timing of AIH onset could not be precisely determined. Logistic regression is well-suited for this cross-sectional risk estimation and is consistent with the intended clinical application as a point-of-care screening and referral-prioritisation tool (20).

### Missing data

Among the 382 patients initially screened, 36 (9.4%) were excluded owing to having had more than 20% missing data among core study variables. In the final analytic cohort of 346 patients, variable-specific missingness rates were as follows: faecal calprotectin 6.1% (21/346), ESR 3.8% (13/346), SMA 2.9% (10/346), PT-INR 2.0% (7/346), and each of the remaining variables having ≤3 missing values (≤0.9%). The complete-case rate was 84.7% (293/346). Missingness was assumed to be at random, based on analysis showing that missing patterns were associated with year of enrolment and referring centre but not with the outcome. Multiple imputation by chained equations (MICE) was performed with 20 imputed datasets and 50 iterations to handle residual missing data. Rubin’s rules were applied to pool parameter estimates and standard errors across imputed datasets. Sensitivity analyses using complete cases only yielded results consistent with the imputed analyses (21).

All statistical analyses were performed using R software (version 4.3.2; R Foundation for Statistical Computing, Vienna, Austria). The rms, glmnet, mice, and pROC packages were used for modelling, variable selection, imputation, and ROC analysis, respectively. A two-sided *p* value <0.05 was considered statistically significant.

## Results

### Baseline characteristics

A total of 346 children with intestinal diseases were included in the analysis, comprising 68 (19.7%) with concurrent AIH and 278 (80.3%) without AIH. The study flow, including the temporal sequence from initial gastroenterology evaluation to hepatological assessment, is presented in Figure 1. The baseline demographic and clinical characteristics of the two groups are shown in Table 1. Children in the AIH group were more likely to be female (64.7% vs. 48.2%, *p* = 0.015) and had longer disease duration (median 18.5 vs. 11.3 months, *p* = 0.002). The distribution of intestinal disease types differed significantly between groups (*p* = 0.008), with a higher proportion of UC in the AIH group (55.9% vs. 35.3%). Among UC patients, pancolitis was significantly more prevalent in the AIH group (73.7% vs. 35.7%, *p* < 0.001). Extraintestinal manifestations (32.4% vs. 17.3%, *p* = 0.005) and family history of autoimmune disease (26.5% vs. 11.5%, *p* = 0.002) were also more common in the AIH group.

**Figure 1:**
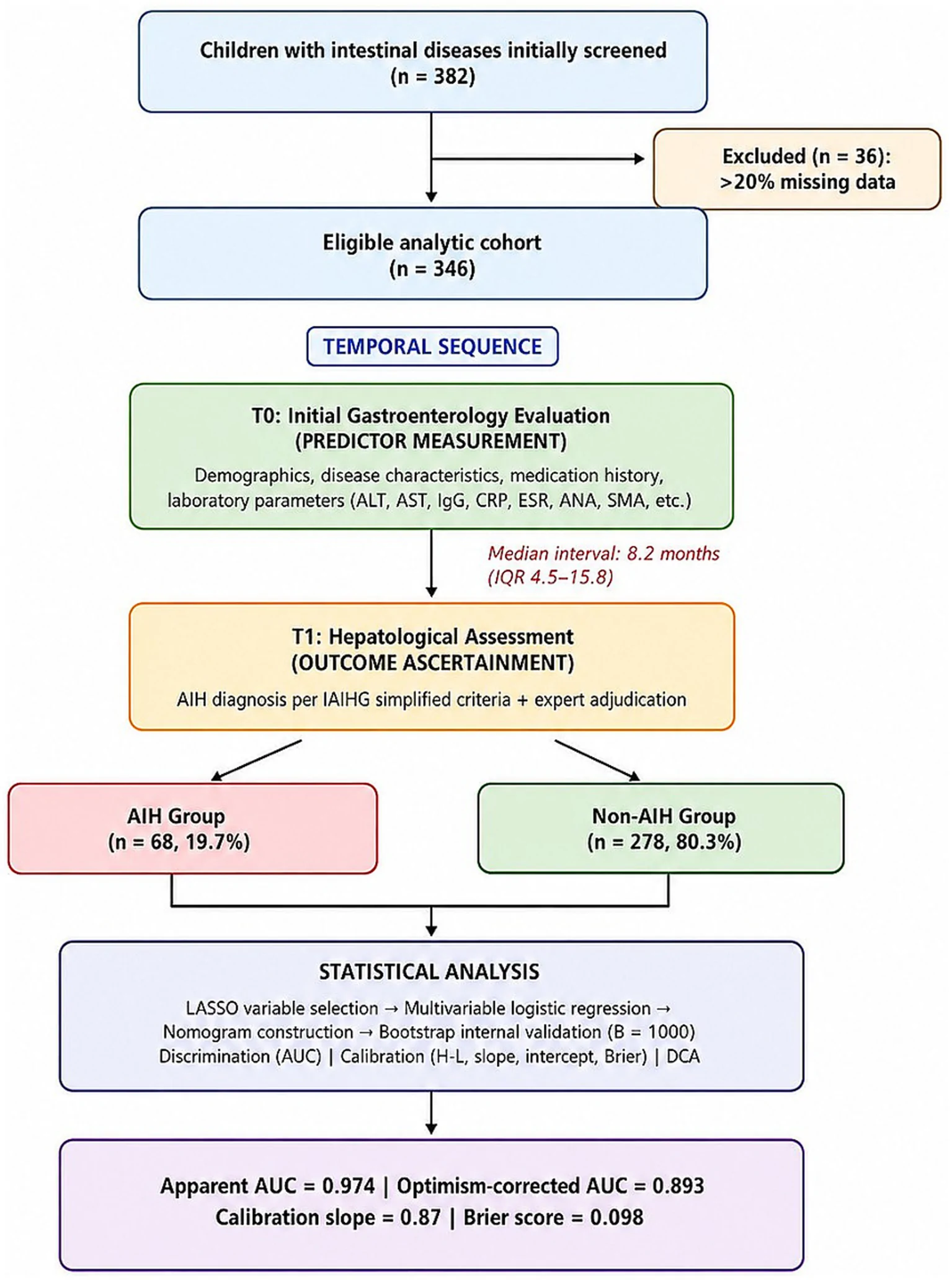
Study flow diagram illustrating participant selection, temporal sequence of predictor measurement and outcome ascertainment, and the analytic framework.

**Table 1:** Baseline demographic and clinical characteristics of the study population.

Variable	AIH group (*n* = 68)	Non-AIH group (*n* = 278)	*p* value
Age (years), median (IQR)	8.4 (5.2–12.1)	7.8 (4.6–11.5)	0.317
Female, *n* (%)	44 (64.7)	134 (48.2)	0.015
BMI *z*-score, mean ± SD	−0.68 ± 1.24	−0.42 ± 1.18	0.108
Type of intestinal disease, *n* (%)			0.008
Ulcerative colitis	38 (55.9)	98 (35.3)	
Crohn’s disease	19 (27.9)	126 (45.3)	
IBD-unclassified	5 (7.4)	22 (7.9)	
Celiac disease	4 (5.9)	18 (6.5)	
Other	2 (2.9)	14 (5.0)	
Disease duration (months), median (IQR)	18.5 (10.2–32.4)	11.3 (5.8–22.6)	0.002
Pancolitis (UC patients), *n* (%)	28/38 (73.7)	35/98 (35.7)	<0.001
Extraintestinal manifestations, *n* (%)	22 (32.4)	48 (17.3)	0.005
Family history of autoimmune disease, *n* (%)	18 (26.5)	32 (11.5)	0.002

### Laboratory parameters

A between-group comparison of laboratory parameters is presented in Table 2 and Figure 2. Hepatic injury and cholestasis-related markers were significantly higher in the AIH group, including ALT (median 168.5 vs. 28.2 U/L, *p* < 0.001), AST (median 145.3 vs. 32.6 U/L, *p* < 0.001), GGT (median 78.4 vs. 22.1 U/L, *p* < 0.001), ALP (median 245.6 vs. 198.4 U/L, *p* = 0.012), and total bilirubin (median 32.8 vs. 12.4 μmol/L, *p* < 0.001), whereas albumin was significantly lower in the AIH group (mean 32.1 vs. 38.5 g/L, *p* < 0.001). Immunological markers were also markedly different, with higher IgG levels (median 21.8 vs. 12.6 g/L, *p* < 0.001), higher rates of ANA positivity (72.1% vs. 12.6%, *p* < 0.001), and higher rates of SMA positivity (58.8% vs. 5.4%, *p* < 0.001) in the AIH group. Inflammatory markers including CRP (median 18.6 vs. 7.2 mg/L, *p* < 0.001) and ESR (median 35.2 vs. 16.8 mm/h, *p* < 0.001) were significantly higher in the AIH group.

**Table 2:** Comparison of laboratory parameters between AIH and non-AIH groups.

Variable	AIH group (*n* = 68)	Non-AIH group (*n* = 278)	*p* value
ALT (U/L), median (IQR)	168.5 (82.3–342.6)	28.2 (16.5–48.7)	<0.001
AST (U/L), median (IQR)	145.3 (68.7–298.4)	32.6 (18.2–52.3)	<0.001
GGT (U/L), median (IQR)	78.4 (35.6–156.2)	22.1 (12.8–38.5)	<0.001
ALP (U/L), median (IQR)	245.6 (168.3–358.2)	198.4 (142.5–285.6)	0.012
Total bilirubin (μmol/L)	32.8 (15.6–68.4)	12.4 (8.2–18.6)	<0.001
Albumin (g/L), mean ± SD	32.1 ± 5.8	38.5 ± 4.2	<0.001
IgG (g/L), median (IQR)	21.8 (16.5–28.4)	12.6 (9.8–15.2)	<0.001
CRP (mg/L), median (IQR)	18.6 (8.4–35.2)	7.2 (3.1–14.8)	<0.001
ESR (mm/h), median (IQR)	35.2 (22.5–52.8)	16.8 (8.4–28.6)	<0.001
Faecal calprotectin (μg/g)	486.2 (245.8–825.6)	285.4 (125.6–548.2)	0.001
PT-INR	1.18 ± 0.22	1.04 ± 0.12	<0.001
ANA positive, *n* (%)	49 (72.1)	35 (12.6)	<0.001
SMA positive, *n* (%)	40 (58.8)	15 (5.4)	<0.001

**Figure 2:**
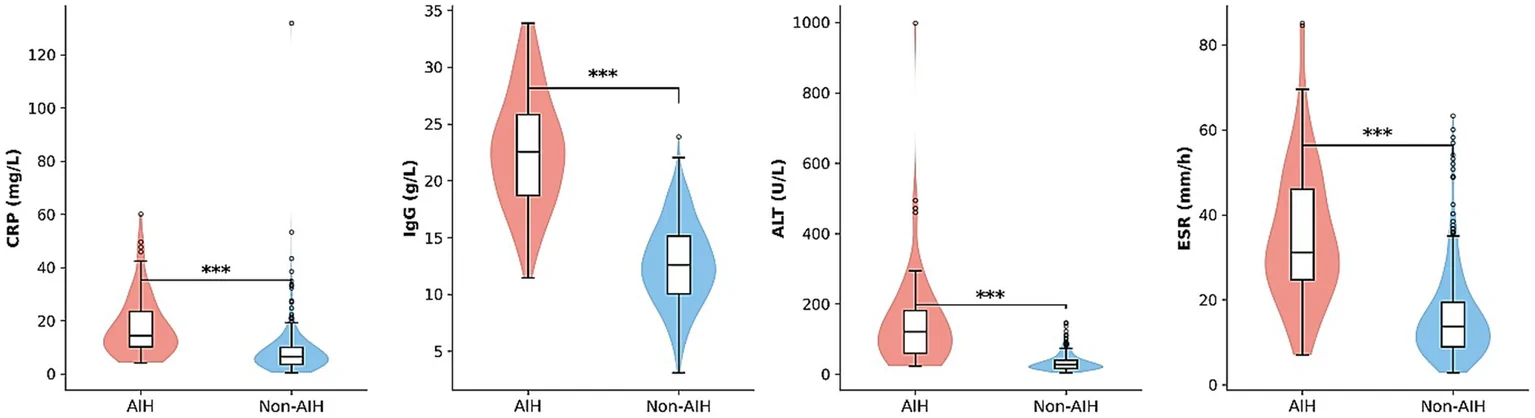
Violin-box plots comparing serum levels of C-reactive protein (CRP), immunoglobulin G (IgG), alanine aminotransferase (ALT), and erythrocyte sedimentation rate (ESR) between the AIH and non-AIH groups. *** indicates *p* < 0.001.

### Medication history

Medication exposures prior to the baseline assessment are compared in Table 3. Systemic corticosteroid use was significantly more frequent in the AIH group (55.9% vs. 32.4%, *p* < 0.001). The use of 5-aminosalicylic acid, thiopurines, methotrexate, biologic agents, and exclusive enteral nutrition did not differ significantly between groups.

**Table 3:** Comparison of medication history between AIH and non-AIH groups.

Medication	AIH group (*n* = 68)	Non-AIH group (*n* = 278)	*p* value
5-ASA, *n* (%)	45 (66.2)	173 (62.2)	0.546
Systemic corticosteroids, *n* (%)	38 (55.9)	90 (32.4)	<0.001
Thiopurines, *n* (%)	33 (48.5)	100 (36.0)	0.058
Methotrexate, *n* (%)	8 (11.8)	24 (8.6)	0.430
Biologic agents, *n* (%)	15 (22.1)	51 (18.3)	0.480
Exclusive enteral nutrition, *n* (%)	12 (17.6)	58 (20.9)	0.556

### Variable selection and multivariable analysis

The LASSO regression identified 8 variables with non-zero coefficients at the optimal *λ*_1se_: disease duration ≥12 months, CRP ≥ 15 mg/L, IgG ≥ 16 g/L, ESR ≥ 25 mm/h, ANA positivity, corticosteroid use, pancolitis, and family history of autoimmune disease. These 8 variables were then entered into a standard multivariable logistic regression model (Table 4). All 8 variables remained independently associated with concurrent AIH: disease duration ≥12 months (OR 2.34, 95% CI 1.28–4.28, *p* = 0.006), CRP ≥ 15 mg/L (OR 3.18, 95% CI 1.76–5.74, *p* = 0.001), IgG ≥ 16 g/L (OR 4.52, 95% CI 2.41–8.48, *p* < 0.001), ESR ≥ 25 mm/h (OR 2.01, 95% CI 1.12–3.61, *p* = 0.019), ANA positivity (OR 3.87, 95% CI 2.05–7.31, *p* < 0.001), corticosteroid use (OR 1.95, 95% CI 1.08–3.52, *p* = 0.027), pancolitis (OR 2.68, 95% CI 1.43–5.02, *p* = 0.003), and family history of autoimmune disease (OR 2.43, 95% CI 1.19–4.96, *p* = 0.014). A forest plot displaying ORs and 95% CIs for all associated variables is shown in Figure 3.

**Table 4:** Multivariate logistic regression analysis of variables associated with concurrent AIH (variables selected by LASSO penalization).

Variable	*β* coefficient	SE	OR (95% CI)	Wald *χ*^2^	*p* value
Disease duration ≥12 months	0.851	0.308	2.34 (1.28–4.28)	7.63	0.006
CRP ≥ 15 mg/L	1.157	0.301	3.18 (1.76–5.74)	14.76	0.001
IgG ≥ 16 g/L	1.508	0.321	4.52 (2.41–8.48)	22.08	<0.001
ESR ≥ 25 mm/h	0.698	0.298	2.01 (1.12–3.61)	5.49	0.019
ANA positive	1.354	0.325	3.87 (2.05–7.31)	17.36	<0.001
Corticosteroid use	0.668	0.301	1.95 (1.08–3.52)	4.92	0.027
Pancolitis	0.986	0.319	2.68 (1.43–5.02)	9.55	0.003
Family history of autoimmune disease	0.888	0.362	2.43 (1.19–4.96)	6.01	0.014

SE, standard error; OR, odds ratio; CI, confidence interval; CRP, C-reactive protein; IgG, immunoglobulin G; ESR, erythrocyte sedimentation rate; ANA, antinuclear antibody. Variables were selected using LASSO (least absolute shrinkage and selection operator) logistic regression with 10-fold cross-validation at *λ*_1se_. The unpenalized ORs and 95% CIs are reported from the standard multivariable logistic regression model using the LASSO-selected variables.

**Figure 3:**
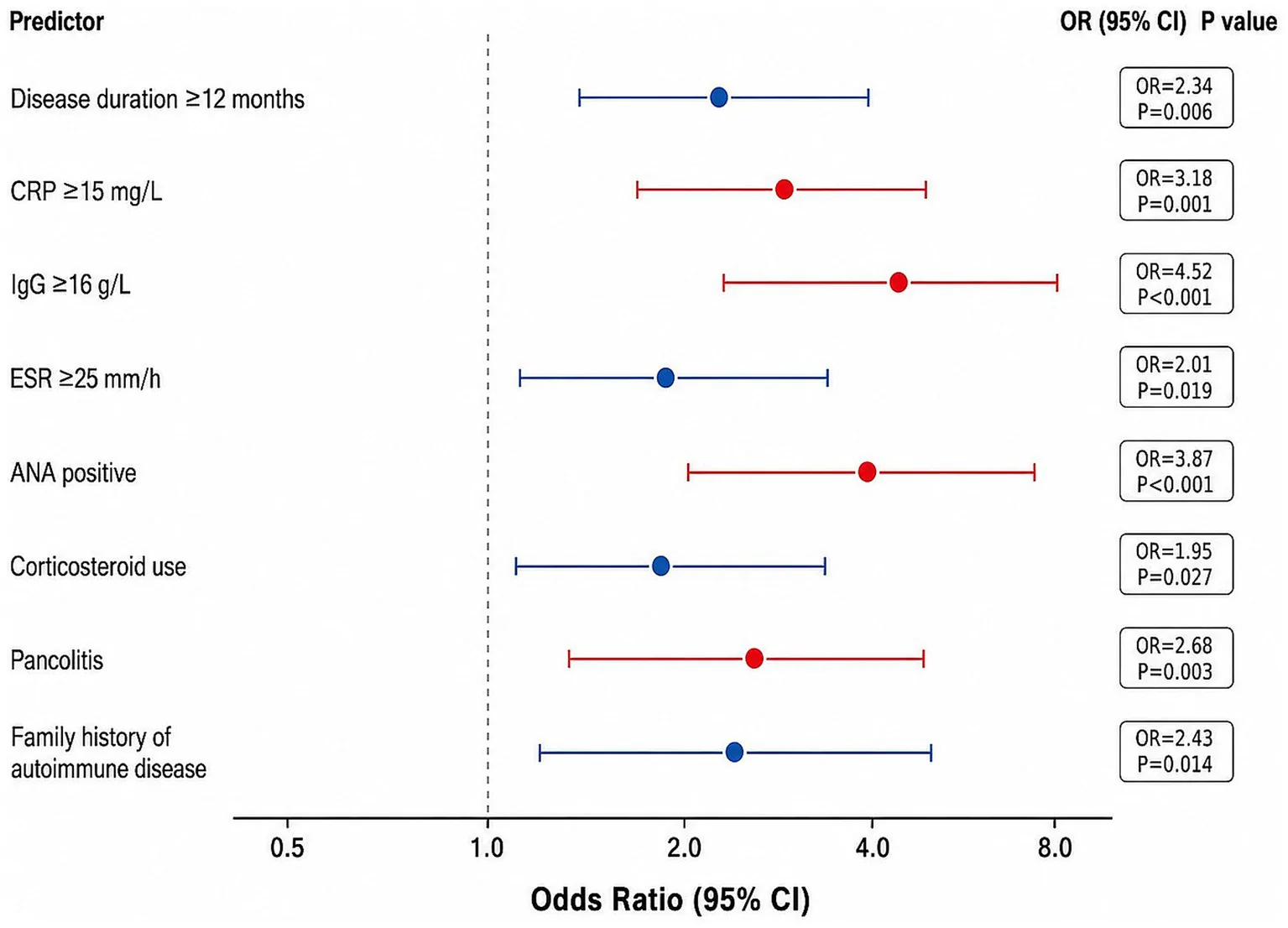
Forest plot displaying odds ratios (ORs) and 95% confidence intervals (CIs) for the eight variables associated with concurrent AIH in the multivariable logistic regression model. Red circles and lines indicate variables with OR > 2.5; Blue circles and lines indicate variables with OR ≤ 2.5.

### Nomogram construction and performance

A nomogram incorporating the 8 variables associated with concurrent AIH was constructed to estimate individualised screening risk, rather than to provide a definitive diagnosis (Figure 4). Each variable contributes a specific point score; the total points were summed and mapped to an estimated probability ranging from 0.05 to 0.95.

**Figure 4:**
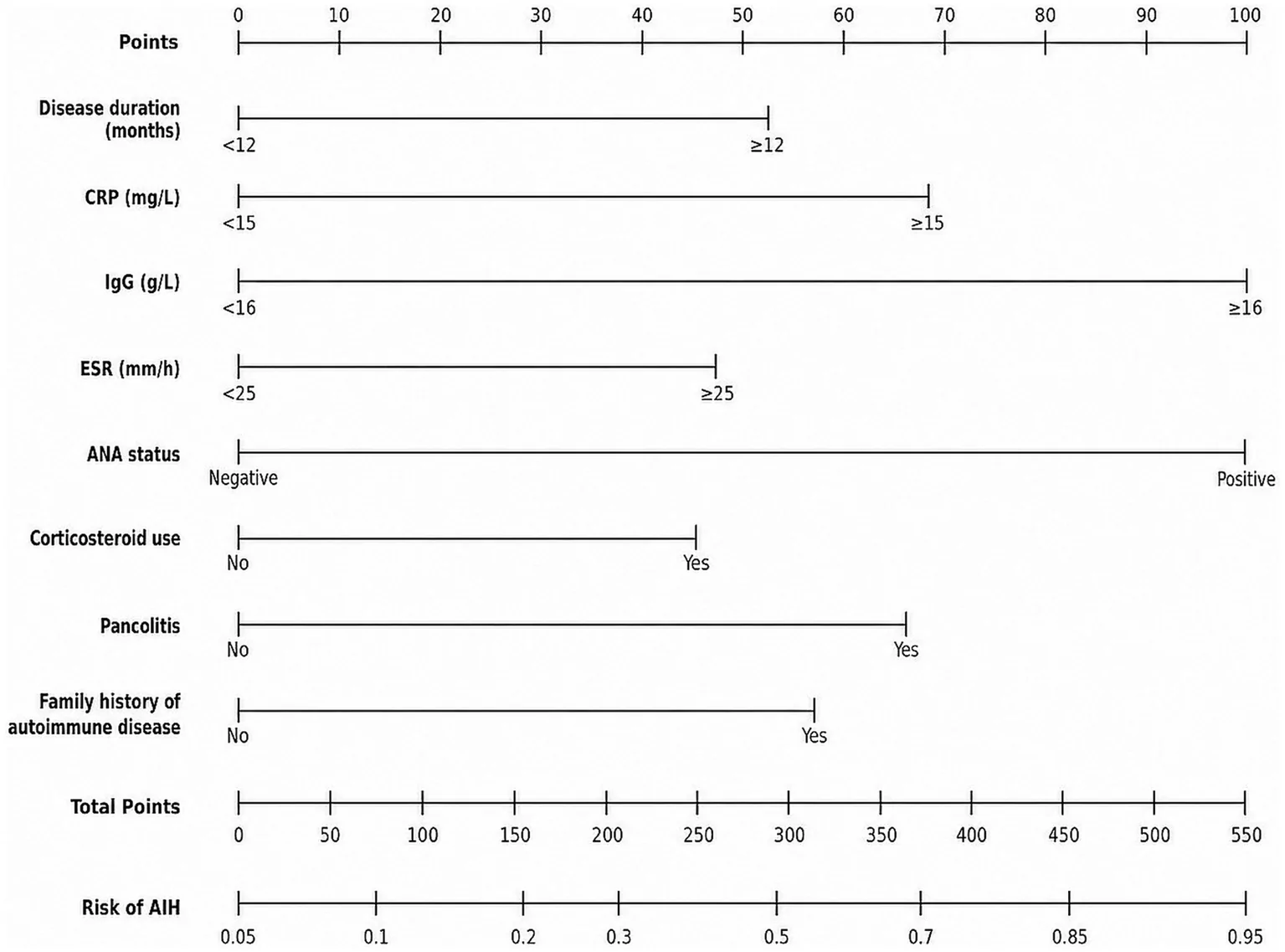
Nomogram for estimating the screening risk of concurrent autoimmune hepatitis (AIH) in children with intestinal diseases. Each variable corresponds to a point value on the top scale; the total points are summed and mapped to the estimated probability on the bottom scale.

The model demonstrated an apparent AUC of 0.974 (95% CI 0.958–0.990) in the development cohort (Figure 5). After 1,000 bootstrap resamples, the optimism-corrected AUC was 0.893 (95% CI 0.852–0.934), with an estimated optimism of 0.081, indicating a moderately optimistic apparent performance owing to the relatively high predictor-to-event ratio. The uniform shrinkage factor was 0.83.

**Figure 5:**
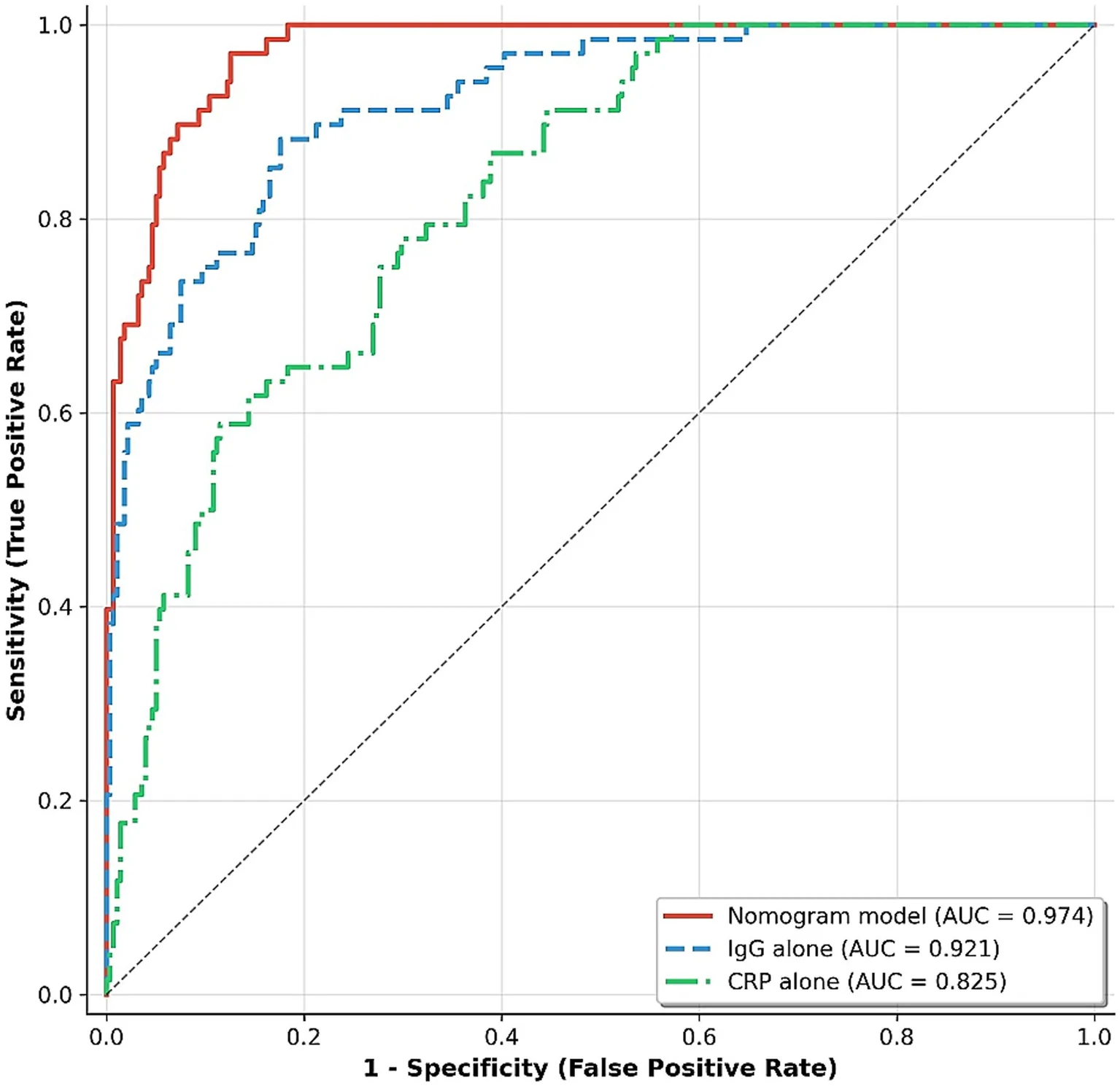
Receiver operating characteristic (ROC) curves comparing the discriminative performance of the screening nomogram (apparent AUC = 0.974), IgG alone (AUC = 0.921), and CRP alone (AUC = 0.825). The reported AUC of 0.974 represents the apparent (training) value; the bootstrap-corrected AUC is 0.893.

Calibration assessment demonstrated good agreement between estimated and observed probabilities. The Hosmer–Lemeshow test was non-significant (*χ*^2^ = 8.37, *p* = 0.398), indicating no evidence of systematic miscalibration. The calibration slope was 0.87 (ideal = 1.0), the calibration intercept was −0.03 (ideal = 0.0), and the Brier score was 0.098. The calibration plot (Figure 6) showed close alignment between estimated and observed probabilities across the full range of risk, with the bias-corrected bootstrap curve closely tracking the ideal calibration line.

**Figure 6:**
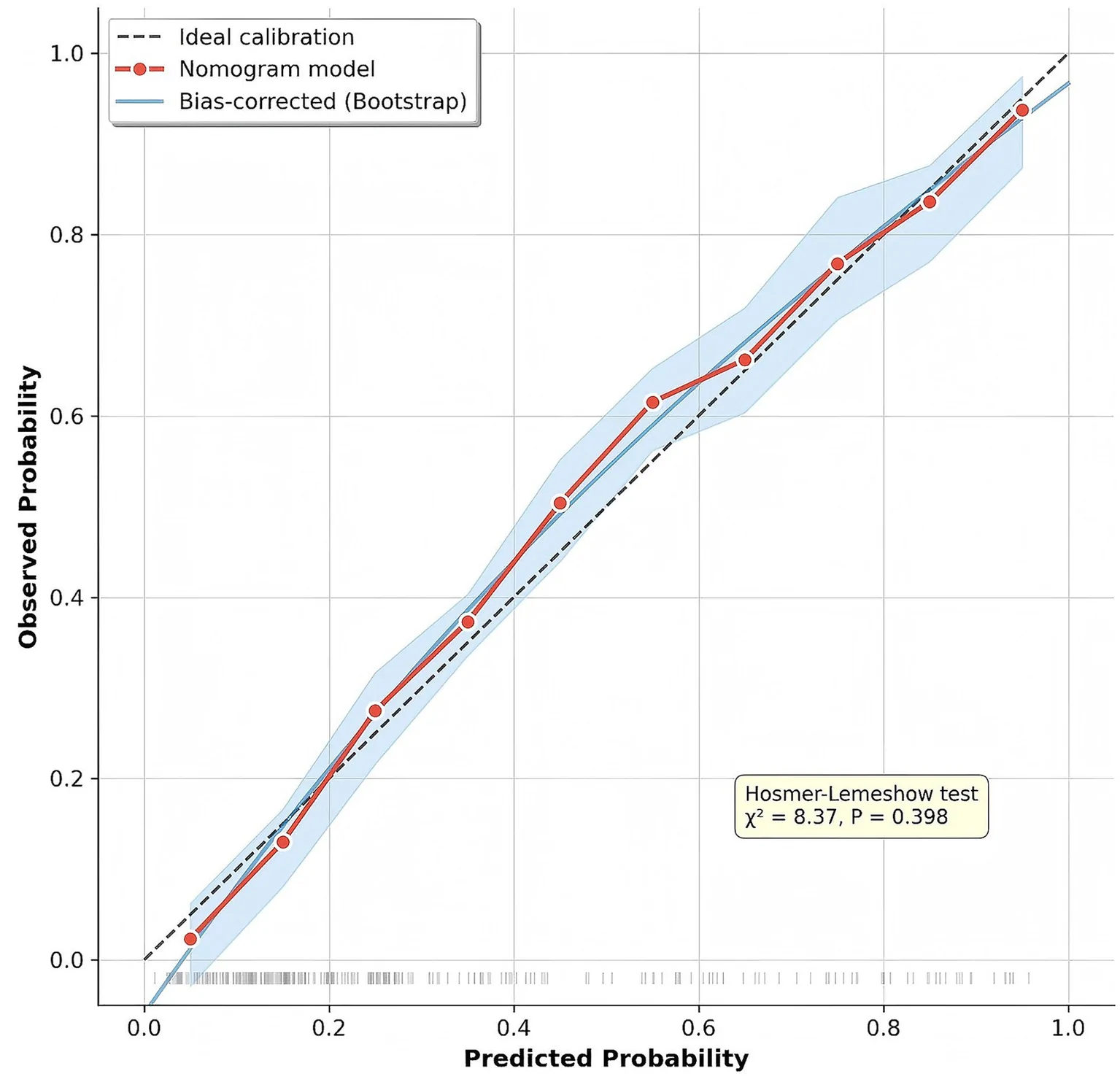
Calibration plot comparing observed and estimated probabilities of AIH. The red line with dots represents the nomogram model, the dashed black line represents ideal calibration, and the blue shaded area represents the bias-corrected bootstrap estimate. The Hosmer-Lemeshow test result (*χ*^2^ = 8.37, *p* = 0.398) is displayed.

Decision curve analysis (Figure 7) demonstrated that the nomogram provided a positive net benefit over the entire range of threshold probabilities tested (0–80%), substantially exceeding both the treat-all and treat-none strategies, as well as the single-predictor CRP model. This indicates that clinical application of the nomogram for screening and referral-prioritisation decisions could increase the number of children appropriately flagged for hepatological evaluation while reducing unnecessary referrals compared with alternative strategies.

**Figure 7:**
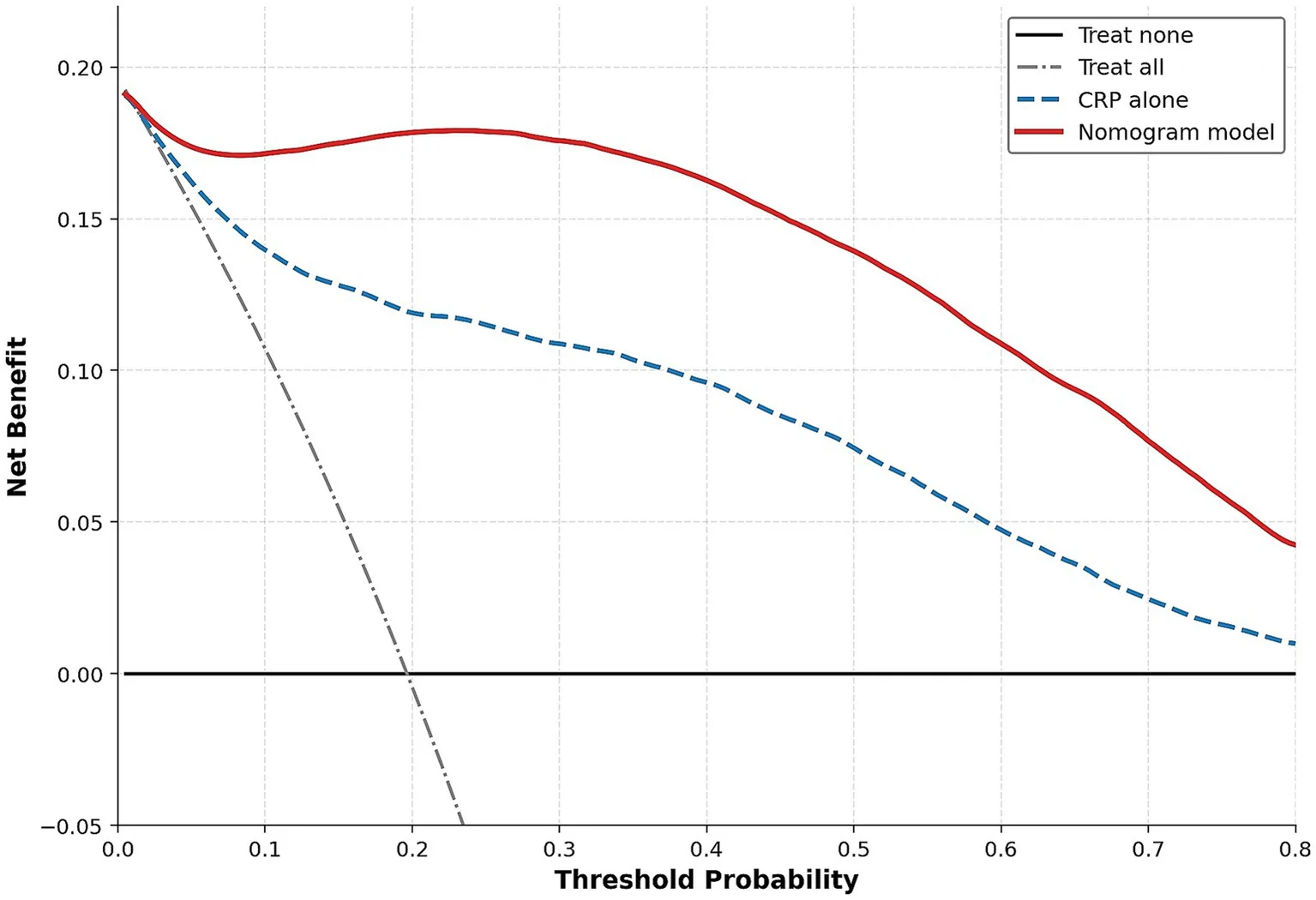
Decision curve analysis (DCA) comparing the net benefit of the screening nomogram, CRP alone, treat-all strategy, and treat-none strategy across a range of threshold probabilities.

## Discussion

This study developed and internally validated a nomogram-based screening and risk-stratification model for identifying children with intestinal diseases who warrant hepatological evaluation for concurrent AIH. The model incorporates 8 readily available clinical and laboratory parameters (disease duration, CRP, IgG, ESR, ANA status, corticosteroid use, pancolitis, and family history of autoimmune disease) and demonstrates a strong discriminative ability with an optimism-corrected AUC of 0.893 after internal bootstrap validation. To our knowledge, this is the first study to develop a clinically applicable screening tool for AIH risk stratification in the paediatric intestinal disease population.

The identification of IgG ≥ 16 g/L as the strongest associated variable (OR 4.52) is consistent with the established role of hypergammaglobulinemia as a hallmark feature of AIH (1, 2). Elevated IgG levels reflect polyclonal B-cell activation and are incorporated into the IAIHG simplified diagnostic criteria (16). Although elevated IgG is itself a component of the AIH diagnostic criteria, it is important to note that in the present model, IgG was measured at the initial gastroenterology evaluation, temporally preceding the formal hepatological workup and AIH diagnosis. At this early stage, IgG elevation may reflect generalised immune dysregulation associated with the underlying intestinal disease, and it should be interpreted as a screening signal rather than as independent diagnostic proof of AIH. The same temporal distinction applies to ANA positivity (OR 3.87), which was assessed as part of the routine autoantibody panel at the initial evaluation. Nevertheless, because both IgG and ANA overlap with components of AIH diagnostic assessment, residual incorporation bias cannot be fully excluded and should be considered when interpreting the model (22).

The association between elevated CRP (≥15 mg/L, OR 3.18) and ESR (≥25 mm/h, OR 2.01) and AIH risk underscores the role of systemic inflammation in hepatic autoimmunity. High levels of systemic inflammation in children with intestinal diseases may reflect more aggressive immune activation that extends beyond the gut, increasing the likelihood of immune-mediated liver injury through the gut–liver axis (5, 6). Persistent inflammatory signalling can enhance antigen presentation in the liver, activate hepatic stellate cells, and disrupt hepatic immune tolerance (7).

Pancolitis was identified as a variable associated with concurrent AIH (OR 2.68), which is consistent with prior reports suggesting that extensive colonic involvement in UC is associated with a higher prevalence of hepatobiliary complications (23, 24). The extensive mucosal surface area affected in pancolitis may lead to greater disruption of the intestinal barrier, facilitating increased translocation of bacterial products and immune mediators to the liver (25). Family history of autoimmune disease (OR 2.43) reflects the contribution of shared genetic susceptibility, particularly in HLA alleles associated with both IBD and AIH (26).

Corticosteroid use was independently associated with AIH risk (OR 1.95). This association likely reflects confounding by indication rather than a direct causal effect: children requiring corticosteroids tend to have more severe or refractory intestinal disease, which itself is associated with greater systemic immune dysregulation and higher risk of concurrent AIH (27). The inclusion of corticosteroid use in the model captures this information as a surrogate marker of disease severity that is readily available in clinical practice.

An important methodological consideration in this study is the relationship between predictor timing and outcome ascertainment. All predictors were measured at the initial gastroenterology evaluation, which preceded the hepatological assessment by a median of 8.2 months (IQR 4.5–15.8 months). The model is, therefore, designed as a screening and referral-prioritisation tool to identify children who should undergo hepatological evaluation at the time of their intestinal disease diagnosis, rather than as a diagnostic algorithm or a model for predicting future *de novo* onset of AIH. This distinction is clinically meaningful because early hepatological referral can lead to timely diagnosis and treatment initiation, potentially preventing progression to advanced fibrosis (10, 11).

Several methodological strengths and limitations merit discussion. Least absolute shrinkage and selection operator penalisation was used for variable selection to mitigate the overfitting risk inherent in traditional stepwise approaches, particularly given the relatively low events-per-variable (EPV) ratio of approximately 8.5 (68 events for 8 predictors). While this EPV is below the commonly recommended threshold of 10–20, bootstrap internal validation with 1,000 resamples was performed to quantify optimism, and the shrinkage factor of 0.83 indicates moderate overfitting (28). The gap between the apparent AUC (0.974) and the optimism-corrected AUC (0.893) of 0.081 further confirms that the model demonstrates some degree of optimism, which is expected in a development cohort. Therefore, the optimism-corrected performance estimates should be emphasised, and external validation remains necessary.

The dichotomisation of continuous biomarkers (disease duration, CRP, IgG, ESR) was performed to facilitate clinical application of the nomogram at the bedside, where binary risk stratification is often more actionable than continuous probability estimation. The selected cutoffs were determined by ROC-based Youden index optimisation and closely aligned with clinically recognised thresholds. However, because data-driven cutoff selection can overfit sample-specific distributions and may lead to optimistic performance estimates, these thresholds should be regarded as provisional screening thresholds requiring external validation. Sensitivity analyses confirmed that models using the continuous forms of these variables produced consistent results in terms of both the direction of association and statistical significance.

This study has several limitations that should be acknowledged. First, the retrospective single-centre design introduces potential selection and information bias, and the findings may not be generalisable to other populations or healthcare settings. Second, only bootstrap internal validation was performed; external validation in independent cohorts from different institutions and geographic regions is essential before this model can be recommended for routine clinical use (29). Third, the relatively small number of outcome events (*n* = 68) limits the statistical power for detecting weaker predictors and increases the risk of model instability, despite the use of LASSO regularisation and bootstrap correction. Fourth, the exact onset of AIH could not be determined in this retrospective cohort, which precluded the use of time-to-event modelling. Prospective studies with serial liver enzyme monitoring could better define the temporal relationship between predictor measurement and AIH development. Fifth, histological data (liver biopsy) were not uniformly available for all patients, and the interface hepatitis component of the IAIHG criteria relied on clinical and biochemical adjudication in some cases (30). Sixth, certain predictors, particularly ANA positivity and elevated IgG, overlap with components of the AIH diagnostic criteria, raising the possibility of incorporation bias. The temporal separation between baseline measurement and formal diagnosis, the inclusion of these variables within a broader multivariable model, and the intended use as a screening rather than diagnostic tool may reduce this concern, but they do not eliminate it (31). Seventh, the ROC/Youden-derived cutoffs were selected within the development cohort and may be unstable across settings with different disease prevalence, referral patterns, or laboratory reference ranges. These thresholds should, therefore, be interpreted as provisional screening thresholds rather than universally applicable diagnostic cutoffs.

## Conclusion

In conclusion, this study presents a nomogram integrating 8 clinical and laboratory parameters for screening and risk stratification of concurrent AIH in children with intestinal diseases. The model demonstrates strong discrimination (optimism-corrected AUC 0.893) and adequate calibration (calibration slope 0.87, Brier score 0.098), with favourable clinical utility suggested by DCA. External validation in multicentre, geographically diverse cohorts is an essential next step to establish the generalisability and clinical readiness of this tool. If validated, this nomogram may serve as a practical screening aid to guide timely hepatological referral in children with intestinal diseases, but it should not replace formal diagnostic assessment for AIH.

## Data Availability

The original contributions presented in the study are included in the article/Supplementary material, further inquiries can be directed to the corresponding author.
