# Supplementary material for: Development and internal validation of a nomogram-based screening tool for concurrent autoimmune hepatitis in children with intestinal diseases: a retrospective cohort study
Source: Front Med (Lausanne). 2026 Oct 7;13:1861363. doi: 10.3389/fmed.2026.1861363 (PMC13683181; doi:10.3389/fmed.2026.1861363)
Supplement: Supplementary file 1 [file Data_Sheet_1.docx]

**Supplementary Table S1.** *Sensitivity analysis using continuous forms of dichotomized variables*

| **Variable (continuous)** | **OR per unit increase** | **95% CI** | **P value** |
| --- | --- | --- | --- |
| Disease duration (per 6 months) | 1.28 | 1.09–1.51 | 0.003 |
| CRP (per 5 mg/L) | 1.35 | 1.18–1.54 | <0.001 |
| IgG (per 2 g/L) | 1.52 | 1.31–1.76 | <0.001 |
| ESR (per 5 mm/h) | 1.18 | 1.06–1.32 | 0.004 |

**Note:** Multivariable logistic regression adjusted for all eight predictors, with disease duration, CRP, IgG, and ESR entered as continuous variables. ANA positivity, corticosteroid use, pancolitis, and family history of autoimmune disease remained as binary variables. All four continuous variables showed consistent direction and statistical significance compared with the dichotomized model (Table 4).
